# New approach to the differentiation of marble samples using thermal analysis and chemometrics in order to identify provenance

**DOI:** 10.1186/1752-153X-8-35

**Published:** 2014-06-07

**Authors:** Tania Gatta, Emanuela Gregori, Federico Marini, Mauro Tomassetti, Giovanni Visco, Luigi Campanella

**Affiliations:** 1 Department of Chemistry, University of Rome “La Sapienza”, P.le. Aldo Moro 5, I-00185 Rome, Italy

**Keywords:** Marbles, Thermal analysis, Chemometrics, Principal component analysis, Wyden-widmann method

## Abstract

**Background:**

The possibility of applying a novel chemometric approach which could allow the differentiation of marble samples, all from different quarries located in the Mediterranean basin and frequently used in ancient times for artistic purposes, was investigated. By suggesting tentative or allowing to rule out unlikely attributions, this kind of differentiation could, indeed, be of valuable support to restorers and other professionals in the field of cultural heritage.

Experimental data were obtained only using thermal analytical techniques: Thermogravimetry (TG), Derivative Thermogravimetry (DTG) and Differential Thermal Analysis (DTA).

**Results:**

The extraction of kinetic parameters from the curves obtained using these thermal analytical techniques allowed Activation Energy values to be evaluated together with the logarithm of the Arrhenius pre-exponential factor of the main TG-DTG process.

The main data thus obtained after subsequent chemometric evaluation (using Principal Components Analysis) have already proved useful in the identification the original quarry of a small number of archaeological marble finds.

**Conclusion:**

One of the most evident advantages of the thermoanalytical – chemometric approach adopted seems to be that it allows the certain identification of an unknown find composed of a marble known to be present among the reference samples considered, that is, contained in the reference file. On the other hand with equal certainty it prevents the occurrence of erroneous or highly uncertain identification if the find being tested does not belong to the reference file considered.

## Background

In previous years efforts were made to develop a sufficiently rapid method for identifying the provenance of the marbles of which archaeometric and artistic finds [1,2] were composed. The experience acquired in recent years both in the kinetic processing of thermal analytical data, e.g. for calculating, for instance, Ea and log A values, and in the targeted use of chemometric techniques, made it possible to obtain the more consolidated results presented herein.

Marble is certainly the best known and the most frequently used stone in sculptural masterpieces owing to its brightness, translucency, ease of working and polishing, and above all the ease which extremely smooth surfaces of great beauty can be obtained. A wide variety of marbles has been available in the Mediterranean basin for over two millennia [3]. The identification of pure white marbles of Greek, Turkish, Spanish, Italian, or other origin [4], as well as their use to fabricate works of art and cultural heritage, was the subject of study by a number of scientists [5,6]. Indeed, a thorough scientific investigation is usually necessary in order to support and complete the work of historians and restorers [7,8] as, once the type of marble used to make the artistic artefacts under study has been identified, an attempt can be made to solve the problems related to restoration and conservation. The classification of the provenance of a marble object is therefore by no means an easy task [9].

In the past numerous methods have been tried to overcome this problem: macroscopic examination, mineralogical and petrographic identification and several instrumental techniques have been used: X-ray diffraction, X-Ray fluorescence spectroscopy, atomic absorption spectroscopy, neutron activation analysis, mass spectrometry and electron spin resonance spectrometry [5,7,10], on the hypothesis that the knowledge of the chemical properties and the composition of the different types of marble enables the identification of the provenance of the mineral of which the marble find is composed and would also facilitate the identification of the provenance of the work of art itself, its assignment to a given historical period, or indeed of the author who created it. The demand for a rapid instrumental chemical analytical method has therefore increased in recent times. On the other hand, various researches have been reported in the literature by several authors [11-15], including also some of the coauthors of the present paper [16,17], showing how chemometrics can be successfully used to process thermal analytical signals for the characterization of different materials. Consequently, in the present study, tests using a rapid instrumental technique alone for marble differentiation and recovery, namely thermal analysis (TG, DTG, DTA) [18,19], together with data elaboration using classical chemometric methods (PCA etc.) [20], were used to come up with a relatively simple method to try to solve this difficult problem.

## Results and discussion

**Figure 1:**
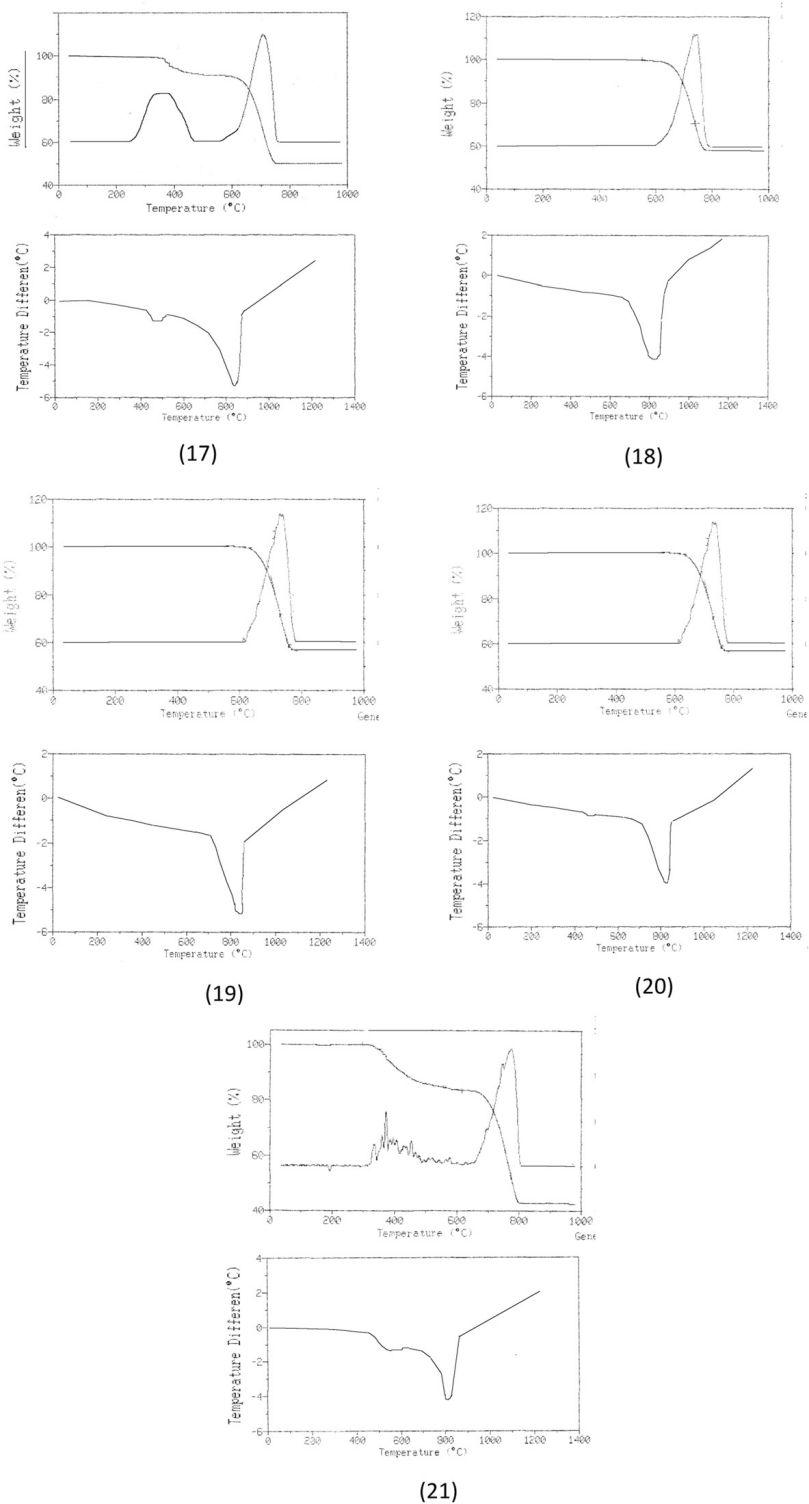
**TG, DTG and DTA curves recorded on the 4 unknown marble samples and those referring to the ‘tempio rotondo’, namely the sample selected as ‘test sample’.** The numbers and marks used to identify the samples in these figures are the same as those used in Table 1, to which reference is made. TG/DTG curves were recorded in the interval 35-1000°C, while DTA curves in the interval 35-1200°C.

All the TG, DTG e DTA curves of all the carefully ground up marble samples tested were recorded under the experimental conditions described in the “Methods” section. Additional file 1: Figure S1 in the supplementary material contains the TG, DTG and DTA curves of the 16 “standard reference marble” samples, while Figure 1 shows the experimental thermal analytical profiles recorded on the 4 unknown marble samples (numbers 18 to 21) and on the “Tempio Rotondo” (identified as number 17) [1].

It is immediately apparent that the observed thermal processes are very limited in number. By far the most significant process is the one observed in the TG/DTG curves, generally between about 700 and 850°C, mainly due to the decomposition of the calcite [1,2], as is confirmed by the corresponding DTA curve, endothermic in a dynamic air atmosphere. In only a few cases, between about 380 and 650°C, a less significant process occurred due to an appreciable dolomite content [1] and thus to the presence of magnesium carbonate in addition to the always preponderant calcium carbonate content; while in CO_2_ atmosphere the endothermic heat of dolomite decomposition can usually split in two signals at higher temperatures [21]. Any other small (hard to detect) mass losses whenever present may be ascribed to the presence of traces of pyrites, graphite, quartz, clay, mica [22], sulphites and gypsum [18].

While the thermogravimetric curves are produced in an air atmosphere, both calcite and dolomite are decomposed in a continuous process between 650 and 850°C [23], while, only by working in a CO_2_ atmosphere, is it possible to clearly distinguish the first decomposition step of the dolomite, which, in this case, takes place at a lower temperature than that of calcite alone [23,24]. However, it is clear that in the low airflow conditions in which the present tests were carried out a certain percentage of CO_2_ was certainly present in the atmosphere over the crucible in which the thermodecomposition processes took place. The temperatures at which marble decomposes are strongly affected not only by the chemical-mineralogical breakdown of the mineral but also by a large number of other properties possessed by the marbles themselves, such as structure, texture and morphological orientation of the grains [22], but also by the grain size, size distribution, grain boundary geometries and crystallographic preferred orientation [25-27]. Moreover, not only does the atmosphere in the crucible (air or CO_2_) have a strong effect on the TG, DTG, DTA curve trends of the marbles, but also the partial pressure of CO_2_ and mechanical grinding plays a significant role regarding the temperature of the main processes observable in these curves [28]. Lastly, also the sample grinding mode [23,29] and the finely ground marble size take on a non secondary importance in determining the trend of the thermoanalytical curves, above all whenever dolomite is present in the marble sample [28]. In such a case the sample grinding mode affects not only the shape of the principal thermal process at about 700 – 850°C, but can also determine the presence or absence of a small decomposition process which can in some cases be observed at lower temperatures (450 – 600°C) [28]. This process has been extensively observed by McCauley et al. [29] and described using the characteristic term “decrepitation”. Indeed it is possible to observe in this connection a typical “swarm” of small consecutive, more or less overlapping, steps, that unfortunately are also difficult to distinguish amid the strong instrumental noise that is inevitably produced in the thermoanalytical curves under these conditions. In practice, therefore, the chemical-physical phenomena associated with the thermal analysis of finely ground marble samples containing dolomite are rather complex. Specific research seems to have ascertained that the dolomite grinding mode concauses significant modification in the solid, involving changes in crystal structure, producing bond breakage, partial decomposition, phase transition and amorphous phase formation [30-32]. However, this complex process still remains to be clarified fully. One explanatory hypothesis could emerge from the tests performed, albeit in different atmospheric conditions compared with the present work, carried out by Caceres et al. [23]. By heating CaCO_3_.MgCO_3_ at 750°C in the presence of CO_2_, these authors initially produced the formation of MgO.CaCO_3_ which subsequently, in the presence of humidity, was probably hydrated to Mg (OH)_2_ + CaCO_3_. In these conditions, in the case of grinding samples, these authors observed a clearcut thermogravimetric process between 500 and 650°C. It may thus possibly be considered legitimate to interpret the latter thermogravimetric step as due to the release of water by the Mg (OH)_2_ under heating, in practice the so-called “structurally bound water”. Returning to the “decrepitation processes” observed also in several of our thermograms at practically the same temperatures, the marble samples tested by us were not previously subjected to heating in a CO_2_ atmosphere as performed by Caceres et al. [23]. However, in this connection, our samples, on being ground up finely and at length in a mortar may have attained relatively high temperatures due to friction. This, in the presence of CO_2_ and atmospheric humidity, may have led to the formation of a non negligible quantity of Mg (OH)_2._. The breakdown of the latter during the subsequent thermogravimetric analysis may well have produced the loss of structurally bound water, with its characteristic series of small successive processes which nevertheless always fall within the same temperature range, namely between 500 and 600°C, which are fully representative of the first thermogravimetric step observed by us in some cases.

As far as the problem of the ‘provenance’ of the marble samples is concerned, over the last few years our research group has tackled this problem on at least two occasions. Initially [1] the approach, not so different to the present one, involved analysing samples of several significant marbles used in ancient times and originating from the Mediterranean basin, but the study was carried out using several different instrumental techniques, such as Atomic Emission Spectroscopy, Coupled Plasma Emission, X-ray diffractometry and Thermal Analysis. The huge quantity of data obtained in this way, processed using classical chemometric methods, evidenced some similarities among the available samples but couldn’t provide conclusive information about possible attributions. In addition, this approach involves the necessity of carrying out a large number of analyses and collecting and classifying huge amounts of data. Recently, our research, carried out for various reasons on different materials of which different archaeological finds and cultural assets were composed and performed using thermal analytical techniques (TG, DTG and DTA) [2], showed that these thermal techniques, above all if the data thereby obtained were suitably processed, can alone solve many problems surrounding the dating or the origin of archaeological material. This led us to carry out further research, described in a previous paper [33], on the problem of the provenance of the marbles using exclusively (but all) the data obtained from the TG curves. Of course, this approach substantially narrows the information down to a single type of data, albeit abundant, as in practice all the raw values making up a thermogravimetric curve were used. For this reason their chemometric treatment was affected by the difficulty encountered in separating the TG data that actually contain information from redundant data that only represent “noise”. An additional problem was represented by the loud instrumental noise that inevitably affects the data obtained using these techniques. Consequently, this meant that the results obtained following this type of approach did not live up to expectations. As we have seen, however, in recent times the thermoanalytical data obtainable by processing the “raw” curves have been considerably refined and have now become much more reliable thanks to the new mathematical processing kinetic methods introduced and the new software used in modern thermoanalytical apparatus. Therefore, in the present study, at first the experimental signals obtained using TG, DTG and DTA thermal analytical techniques were collected and digitalized (see Data processing and chemometrics section). From these, we extracted the values referring to true peak temperatures and mass variations of the main thermogravimetric steps and TG residues at 1000°C (see TG and DTG curves of studied samples in Figure 1). Subsequently, to these values were added the values of the principal DTA peak temperature and above all the activation energy Ea and the log A (A = Arrhenius pre-exponential factor) values of the principal TG-DTG thermal step, evaluated by processing thermogravimetric data using the so-called Wyden and Widmann method [34].

These data, which are displayed in Table 1, constitute the minimum set of variables that can be extracted from the thermal profiles in order to characterize the marble samples in order to differentiate them.

**Table 1:** Thermal analytical data

**Sample number**	**Sample description**	**Abbreviation**	**1st step**		**2nd step**				**Residue at 1000°C**	**DTA**
			**1st T**_**peak**_	**1st loss%**	**2nd T**_**peak**_	**2nd loss%**	**E**_**a**_	**logA**		**2nd T**_**peak**_
1	naxos	naxos		0.00	746.0	43.42	83.86	8.25	55.65	831.0
2	turkish white	turk.w		0.00	746.0	44.56	99.65	10.20	54.14	836.5
3	paros marathi	paros m.	388.5	1.75	720.5	43.37	98.22	10.33	54.44	837.5
4	pentelic	pentelic	382.5	9.30	716.0	40.50	86.41	8.92	49.06	824.5
5	altissimo michelangelo	alt. Michel.		0.00	746.0	43.19	144.92	15.78	55.42	825.4
6	lasa	lasa		0.00	744.0	43.24	78.70	7.67	56.07	865.0
7	aphyon	aphyon		0.00	721.0	44.35	79.93	7.99	54.64	836.0
8	statuario carrara	stat.car.	471.0 (394)	2.06	726.5	42.58	85.54	8.70	54.23	846.0
9	stauario calocara	stat.calo.	365.0	1.55	784.5	41.13	208.28	22.62	55.09	874.5
10	piastra ravaccione	piastra rav.	447.0	1.52	739.0	43.53	121.71	13.04	54.83	818.0
11	altissimo falcucci	alt. Falc.		0.00	708.0	43.68	110.11	12.00	54.84	830.5
12	marmara white	marm.w	300.0	1.37	729.0	43.63	84.51	8.51	54.64	837.0
13	marmara gray	marm.g		0.00	729.5	41.44	84.10	8.49	55.67	834.0
14	carrara colonnata	car.col.		0.00	742.0	43.53	137.84	14.98	54.83	825.5
15	marmo thasos2	thasos	423.5	5.44	725.5	41.56	86.79	8.84	48.25	825.5
16	aphrodis, as white	aphrodis		0.00	724.0	44.35	72.40	7.07	54.64	821.5
17	(a) tempiorotondo	tempiorot.	382.0	8.90	707.5	40.20	80.85	8.28	49.80	841.5
18	(b) It national museum	it.NM1		0.00	735.5	40.80	102.18	10.61	57.60	819.0
19	(c) It national museum	it.NM2		0.00	726.0	40.40	117.36	12.68	57.40	836.0
20	(d) It national museum	it.NM3	494.0	3.30	740.0	39.60	113.48	12.00	52.00	820.5
21	(e) It national museum	it.NM4	418.0	9.50	734.0	40.00	208.71	23.99	49.20	841.0

Legend:

1st T_peak_ – Peak temperature of the 1st step.

1st loss% – Per cent mass loss at the 1^st^ step.

2nd T_peak_ – Peak temperature of the 2nd step.

2nd loss% – Per cent mass loss at the 2nd step.

Ea – Activation energy of the 2nd step.

logA – Logarithm of the Arrhenius pre-exponential factor for the 2nd step.

Residue at 1000°C – Per cent residue at 1000°C.

DTA 2nd peak – DTA peak temperature of the 2nd step.

it.NM1 – Italian National Museum (finding #1).

it.NM2 – Italian National Museum (finding #2).

it.NM3 – Italian National Museum (finding #3).

it.NM4 – Italian National Museum (finding #4).

**Figure 2:**
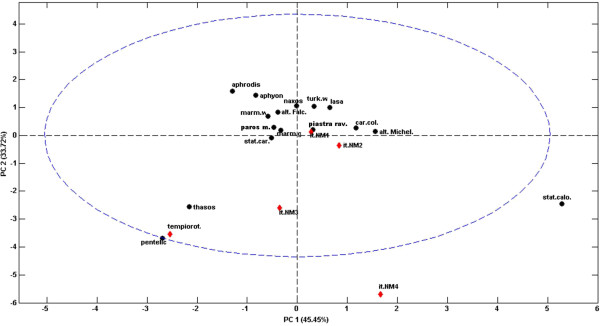
**Principal component analysis on the data reported in Table**1**: Scores plot.** Legend: ● reference samples; (red diamond) unknown samples.

**Figure 3:**
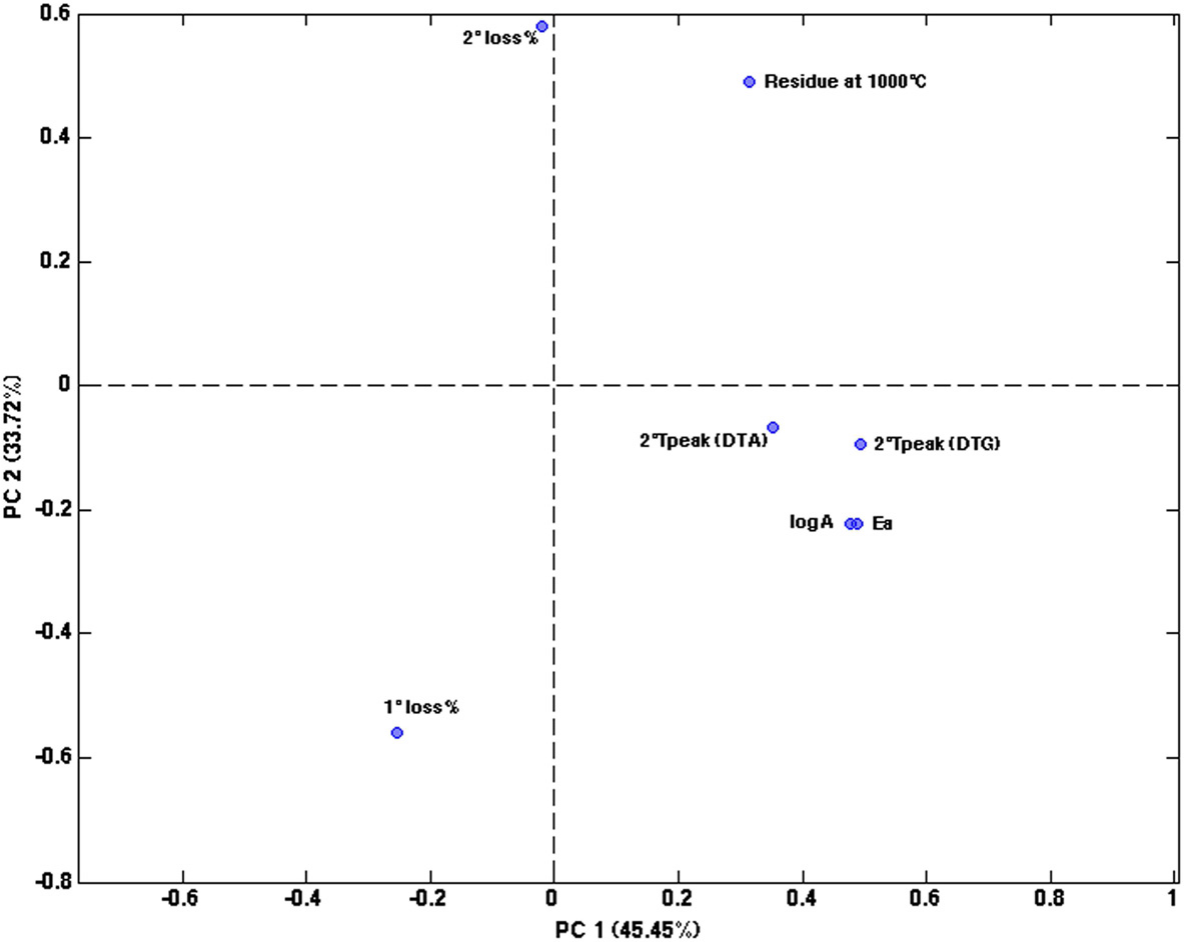
**Principal component analysis on the data reported in Table**1**: Loadings plot.**

To investigate the relations and the similarities among the different marbles, principal component analysis [20] was applied to all the data reported in Table 1 after autoscaling (excluding of course those in the “1st T_peak_” column, which could not be used as no value is included in this column which corresponds to the cases in which this step was not highlighted). Two components explaining about 80% of the original variance were retained as significant, as estimated by cross-validation [35]. The projection of the scores of the known marbles onto the space of the two significant components is reported in Figure 2.It is evident from the figure that most of the samples of known origin fall relatively close to one another in the scores plot, which suggests that they have similar characteristics. Indeed, the marbles which appear to differ more substantially from the rest are Pentelic, Thassos and Statuario Calocara. Inspection of the loadings for the two PCs (see Figure 3) provides an interpretation of these observed differences. In particular, Thassos and Pentelic appear to have a higher mass loss corresponding to the 1st peak and a lower residue at 1000°C, while Statuario Calocara has a higher activation energy.

**Figure 4:**
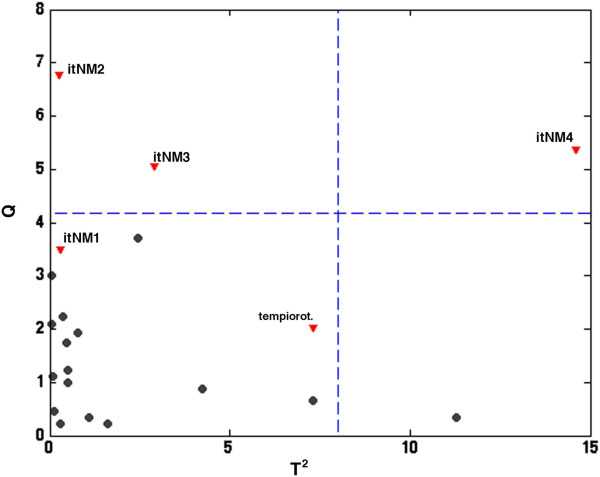
**Principal component analysis on the data reported in Table**1**: T**^**2 **^***vs *Q plot, for outlier detection.** Legend: ● reference samples; (red inverted triangle) unknown samples.

Once the PCA model had been calculated, the 5 unknown samples taken from “tempio rotondo” and from the Italian national museum were projected onto the PC space in order to assess which of the marbles of known origin they were most similar to. (Dis) similarity was evaluated by calculating the Euclidean distance in the score space. Observing the projection in Figure 2, two samples (“Tempio Rotondo” and itNM1) are seen to fall very close to known marbles (Pentelic and Piastra Ravaccione, respectively), while all the remaining ones are mapped onto “empty” regions of space. Moreover, both the T^2^ and the Q statistics [36], which are normally used to detect outlying samples, suggest that the same three samples (itNM2, itNM3, itNM4) are not satisfactorily described by the model and are probably something else (Figure 4). Moreover, the excellent identification of the ‘Tempio Rotondo’ sample as Pentelic, as expected, proves that the method is useful for identifying the marble’s provenance.

### Experimental

#### Samples tested

The 16 marble samples used for reference purposes in the present work were taken directly from their respective quarries with the aid of archaeologists. For the sake of simplicity they have been classified by country of origin.

In addition the marble sample (a) taken from the “tempio rotondo” in Rome (Italy) was used as a “test sample” to verify the validity of the chemometric “test file” used, as it is a known fact that the marble of which it is composed is Pentelic marble.

Lastly four unknown marble samples ((b),(c),(d),(e)), subjected to typing, were taken from archaeological finds conserved in the Rome National Museum, all dating back to the Imperial period (2nd – 3rd A.D.).

#### Thermal Analysis

For TG, DTG and DTA analysis a Dupont TGA 50 thermobalance and a Dupont Instrument base and 1200 DTA Cell, both connected to a PC running Du Pont 2000 data processing software (Du Pont Inc. USA) were used.

About 15 mg of gently and finely ground marble specimens were subjected to thermal analysis on a platinum plate heated over a range of 35-1000°C (TG/DTG) or 35-1200°C (DTA), at a heating rate of 10°C min^−1^ in an airflow of 50 mL min^−1^.

## Conclusion

In conclusion, thermal analysis coupled to principal component analysis provides a valuable tool for differentiating ancient marbles, evidencing the similarities and dissimilarities among the investigated samples. In particular, it allows the differences between a set of marbles chosen as references to be highlighted and interpreted in terms of the experimentally observed thermal transition. Moreover, since the main aim is to verify whether unknown samples could be attributed to any of the marble types chosen as reference, the proposed chemometric approach reported in this investigation seems to have several advantages with respect to the previously described approaches [1,33]. First of all, the consistency between the attribution of the only known test sample and its real origin suggests that the proposed approach is likely to provide accurate results. Additionally, the possibility of using standard diagnostics for the identification of outlying samples proved to be particularly useful in detecting marble samples which do not correspond to any reference material and therefore prevents incorrect interpretations and assignments being made.

Although these results are very promising, they still can’t be considered to be conclusive, as they suffer from the lack of a sufficient number of validation samples of known origin (here, only a single sample was available, and so there was no way of univocally assessing whether the identification of some of the unknown samples as outliers with respect to the reference data, based on the values of Q and T^2^, matched their true origin or not). In general, limited availability of samples of known origin, or reversely the lack of the information on the true origin of available specimens, constitutes the main drawback of the proposed approach.

## Methods

### Data processing and chemometrics

Different softwares were used, first to obtain curves, later for interpolation, smoothing and calculation of peaks and inflection points. In fact, several TGA graphs were registered only by printer connected to the thermobalance, so that, to digitalize these curves, WinDig software (freely downloadable from http://www.unige.ch/sciences/chifi/cpb/windig.html) was used. After this processing step, data values were obtained; however, since the temperature scale was sometimes a little different from graph to graph, data recalculation and homogeneisation was therefore necessary. Using XLCXtrFun (Advanced Systems Design and Development, Red Lion, PA; freely downloadable from http://www.xlxtrfun.com/XlXtrFun/RegisterXlXtrFun.htm) and Approximator *(*freely downloadable from *http://aproxim.sourceforge.net/)* softwares*,* it was then possible to interpolate the raw data using a second order polynomial, 2 sides, method, and to to obtain the same scale for all graphs. The Du Pont 2000 data processing software (Du Pont Inc. USA) was used to extract from the thermoanalytical curves the values of peak temperatures and mass losses.

Finally for Principal Component Analysis, some in-house routines written in Matlab (release 2012b; The Mathworks, Natick, MA) were employed.

### Determination of activation energy and pre-exponential factor using the Wyden-Widmann method

The activation energy values of the main marble’s thermal decomposition process were always obtained starting from the TG and DTG data, which were however processed by means of the multiple linear regression method proposed by Wyden-Widmann [34]. Using this method both the activation energy E_a_ and the log A values of the principal marble’s decomposition step were calculated on the basis of the values derived from a single thermogravimetric test carried out at a constant heating rate, using the multiple linear regression method after logarithmic transformation of the following Arrhenius type equation:

(1) $$\frac{\mathrm{d\alpha}}{\mathrm{dt}}=Ae^{-\frac{E_{a}}{\mathrm{RT}}}{\left(1-\alpha \right)}^{n}$$

where *n* is the order of reaction.

By applying the least squares method, the sum of the squares of the differences between the d/d*t* values calculated using equation (1) and those derived from TG/DTG measurements (taking into account that *α* is calculated using equation (2)

(2) $$\alpha =\frac{\mathrm{\Delta m}}{\Delta m_{\mathrm{tot}}}=\frac{m_{i}-m_{T}}{m_{i}-m_{f}}$$

(where *m*_*i*_ and *m*_*f*_ are the initial and final sample mass and m_T_ is the sample mass at a given temperature and d*α*/d*t* = (d*m*/d*t*)/* Δm*_tot_, where d*m*/d*t* is the rate of mass loss and *Δm*_tot_ = *m*_*i*_-*m*_*f*_) attains its minimum value for given values of *A*, *n* and *E*_a_, which are thereby identified. In particular, the value of the activation energy *E*_a_ is thus determined.

## Abbreviations

TG: Thermogravimetry; DTG: Derivative thermogravimetry; DTA: Differential thermal analysis; PCA: Principal component analysis.

## Competing interests

The authors declare that they have no competing interests.

## Authors’ contributions

MT and LC designed the study; MT and EG performed the TG analysis; FM and GV were in charge of the chemometric data processing; TG contributed to manuscript writing and took care of Figures, Tables and other graphical aspects. All authors read and approved the final manuscript.

## Supplementary Material

Additional file 1: Figure S1 Contains the TG, DTG and DTA curves recorded on the 16 “Standard Reference Marble” samples. The numbers and marks used to identify the samples in these figures are the same as those used in Table 1, to which reference is made. Click here for file
